# New damage model for simulating radiation-induced direct damage to biomolecular systems and experimental validation using pBR322 plasmid

**DOI:** 10.1038/s41598-022-15521-y

**Published:** 2022-07-05

**Authors:** Jinhyung Park, Kwang-Woo Jung, Min Kyu Kim, Hui-Jeong Gwon, Jong-Hyun Jung

**Affiliations:** Advanced Radiation Technology Institute, Korea Atomic Energy Research Institute, Jeongeup, 56212 Republic of Korea

**Keywords:** Biophysics, Biotechnology, Physics

## Abstract

In this work, we proposed a new damage model for estimating radiation-induced direct damage to biomolecular systems and validated its the effectiveness for pBR322 plasmids. The proposed model estimates radiation-induced damage to biomolecular systems by: (1) simulation geometry modeling using the coarse-grained (CG) technique to replace the minimum repeating units of a molecule with a single bead, (2) approximation of the threshold energy for radiation damage through CG potential calculation, (3) calculation of cumulative absorption energy for each radiation event in microscopic regions of CG models using the Monte Carlo track structure (MCTS) code, and (4) estimation of direct radiation damage to biomolecular systems by comparing CG potentials and absorption energy. The proposed model replicated measured data with an average error of approximately 14.2% in the estimation of radiation damage to pBR322 plasmids using the common MCTS code Geant4-DNA. This is similar to the results of previous simulation studies. However, in existing damage models, parameters are adjusted based on experimental data to increase the reliability of simulation results, whereas in the proposed model, they can be determined without using empirical data. Because the proposed model proposed is applicable to DNA and various biomolecular systems with minimal experimental data, it provides a new method that is convenient and effective for predicting damage in living organisms caused by radiation exposure.

## Introduction

The mechanistic modeling of the radiation track structure in the cell nucleus is an efficient way to simulate the complex reaction pattern between DNA and ionizing radiation, which is a major contributor to biological effects following radiation exposure. This modeling method is applied to the Monte Carlo technique, to obtain a Monte Carlo track structure (MCTS) code. The MCTS code has recently been applied to radiation protection in the field of medicine, nuclear energy, and space exploration, and is used as a tool to analyze radiation damage to living cells at the DNA level^1–9^.

Geant4-DNA^10–12^ is a typical MCTS code that has been widely used in recent studies. It calculates the radiation absorption energy in microscopic regions of DNA by modeling particle trajectories as defined by physical interactions for which water is a medium. It is generally considered that radiation absorption energy induces radiation damage through ionization and excitation under certain conditions. Specifically, direct DNA damage can occur through the transfer of charges because of ionization, or a resonance effect that can alter the DNA structure can be created through dissociative attachment^13^. However, the radiation absorption energy as a simulation result does not necessarily correspond to direct damage of DNA molecules. In addition, there is a deficiency of data required to enhance the understanding of the DNA damage process according to radiation doses. Therefore, a damage model based on absorption energy patterns is assumed in mechanistic simulations for estimating radiation-induced DNA damage^14^.

Current damage models calculate the cumulative absorption energy for each radiation event in the "backbone" region of the double helix DNA structure (which is known to be sensitive to radiation), and compares it with a predefined threshold energy to estimate single-strand break (SSB) and double-strand break (DSB) yields as functions of linear energy transfer (LET)^15^. Consequently, the simulation results are closely related to parameters such as radiation absorption and threshold energies. Therefore, it is necessary to specify appropriate initial values for these parameters while constructing a damage model.

The values of threshold energy commonly used in previous studies are 10.79 eV^16–18^ (which is equal to the ionization energy of water) and 17.5 eV^19–21^ (which has been verified through experiments on Auger electrons and I-125)^22–25^. A linear damage model was also proposed in the PARTRAC simulation. In this model, the threshold energy increased from 5 to 37.5 eV based on the adjustment of parameters using experimental data^5,9,22,23^. Meylan et al.^6^, Tang et al.^8^, Lampe et al.^7,25^, and Sakata et al.^9,15^ used the threshold energy calculated in this manner to analyze the contribution to DNA damage of complex structures. In addition, simulations effectively replicated experimental results as functions of LET in all the cases. However, the reliance on experimental data is a limitation of the current standard for determining threshold energy. The insufficiency of experimental data prevents one from defining the initial value of threshold energy for evaluating the radiation resistance of a biomolecular system with a molecular structure different from that of DNA. This makes the radiation absorption energy calculated by simulation meaningless.

In this work, we aimed to mitigate the limitation of existing models by constructing a new damage model that can approximate the initial parameter values without using empirical data. The new damage model is based on a simulation geometry that uses the coarse-grained (CG)^26^ technique, which replaces the minimum repeating units of a biomolecular system with a single bead. The CG volume was used as a unit volume for calculating cumulative radiation absorption energy with the MCTS code. Furthermore, the potential energy of the CG volume was used as the threshold energy for radiation damage. In this study, the new damage model was applied to the estimation of radiation damage in pBR322 plasmids using Geant4-DNA. In addition, its effectiveness was validated by comparing the results to measured data. The uncertainties reported in this paper refer to one standard deviation of mean.

## Methods

### Coarse-grained (CG) geometry modeling: pBR322 plasmid

**Figure 1:**
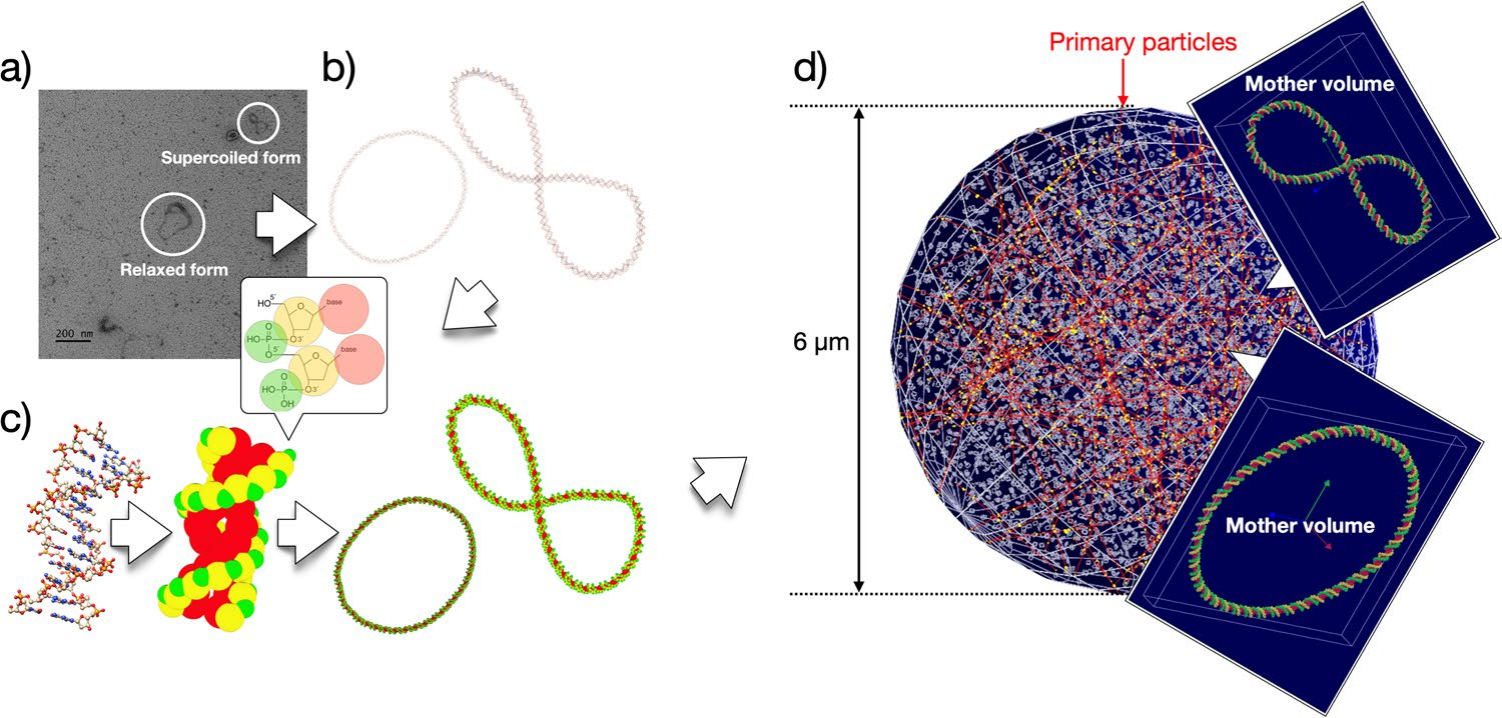
Schematic representation of a CG modeling process for pBR322 plasmid. (**a**) Negative TEM image with 0.2 M NaCl. (**b**) 436 bp sized plasmid atomic model realized with the TEM image. (**c**) Plasmid model generated by transforming an atomic model into a CG model in which each nucleotide is represented by three interaction sites corresponding to the phosphate (green), deoxyribose (yellow), and base (red). (**d**) The test geometry consists of a sphere with 3 μm radius filled with 5,400 individual plasmid segments (Geant4 Qt viewer).

We analyzed the morphological properties of pBR322 samples using 30,000 × magnified negative transmission electron microscopy (TEM) images to model a realistic plasmid structure. pBR322 plasmids at a concentration of 480 ng/μl were dissolved in a TE buffer (10 mM Tris–HCl pH 8.0, 1 mM EDTA). A sample solution of 5 μl was loaded into a carbon film-coated TEM grid. After 90 s, the excess sample amount was removed using distilled water. For negative staining, 5 μl of 1% uracil acetate was used to treat the grid for 1 min. The remaining staining solution was removed using a filter paper. The prepared samples were imaged at 120 kV using a Tecnai G^2^ Spirit electron microscope (FEI; Korea Basic Research Institute, Ochang, South Korea) equipped with lanthanum hexaboride (Lab6). The captured images were recorded using an Ultrascan 4000 charged-coupled device (CCD) camera (Gatan). To analyze the complex structure of the plasmids, variations in the plasmid structure were observed by altering the NaCl concentration to 0.2 M and 1.0 M. Figure 1a shows the pBR322 image obtained under the 0.2 M NaCl concentration. Relaxed circular forms and supercoiled forms were mainly observed. Meanwhile, figure-eight forms with one twist were mostly observed among the supercoiled forms. To model the plasmids with a structure identical to that analyzed using negative TEM, three-dimensional plasmid atomic models in the relaxed and supercoiled forms were sketched using the open-source program Graphite-LifeExplorer^27^. The size was scaled to 1/10 at 436 base pair (bp) to ensure calculation efficiency. Finally, the sketched atomic structure was stabilized using the “minimize structure” tool of the Chimera^28,29^ program. Figure 1b shows the plasmid atomic structure modeled through this process.

For the atomic model in Fig. 1b, it is difficult to evaluate whether a radiation reaction site falls within the range that causes direct radiation damage to DNA. To address this problem, the DNA atomic model was transformed into a CG model using a method of substituting an atomic cluster in close proximity with a single bead^30^. Figure 1c illustrates the process of transforming the DNA atomic model into a CG model. The nucleotides of DNA were realized in the CG model in spherical forms represented by phosphate, deoxyribose, and base. This reduced the reaction sites between DNA and radiation to three per nucleotide. In this case, the initial radius (r) of each sphere was calculated by considering the union volume of the atomic cluster based on the van der Waals radius. The union volumes of phosphate, deoxyribose, and base were evaluated to be 0.050, 0.084, and 0.104 nm^3^, respectively. The radii of each volume calculated by $r=\sqrt[3]{3V/4\pi }$ (V = union volume of atomic cluster) were 2.3, 2.7 and 2.9 Å (hereafter referred to as VDWR) for phosphate, deoxyribose, and base, respectively. Figure 1c shows the result of transforming the atomic model into a CG model. Green, yellow, and red spheres indicate the CG volumes corresponding to phosphate, deoxyribose, and base, respectively. This work was performed by using the MATLAB, and the CG geometry was saved in the same format as the geometry file created by the DNAFabric tool. The DNA CG geometry file was then loaded into the Geant4 simulation space by applying the DNAParser class provided in the Geant4 dnadamage1 example. The overlapping part between CG volumes was removed using the CreateCutSolid() function of the DNAParsar.cc code. Figure 1d shows the plasmid structure imported into the Geant4 simulation space.

### Calculation of CG potential and approximation of threshold energy

The potential energy of CG models reflects the bonding strength of the constituent atomic clusters. Therefore, we determined that the absorbance of radiation energy exceeding the potential energy in the scoring volume would affect the bonding between atoms and thereby, cause radiation damage such as a broken CG model. In addition, we developed a method for replacing the CG potential with threshold energy. The double helix DNA molecular structure has covalent bonds except for the hydrogen bonds between nucleobases. Therefore, the potential energy of the CG volume is mostly occupied by the covalent bonding of the constituent atoms. However, atoms that are not covalently bonded can also partially contribute to the total potential energy of CG volume through electromagnetic force depending on the diatomic distance. Therefore, the potential energy of the CG volume must be carefully calculated considering the contributions of both the bonded and non-bonded atoms. For example, in the case of a phosphate group consisting of PO_4_, because the phosphorus and four oxygen atoms share electrons, they can be considered as bonded. In contrast, oxygen atoms are not covalently bonded to each other, and these diatomic elements can be selected as non-bonded atoms (see Fig. S2).

The total potential energy (U_total_) of a CG model composed of N atoms (i = 1, 2, 3,…N) is generally expressed by the following equation:

1 $${U}_{total}=\stackrel{N}{\sum_{i<j}}{U}_{1}({r}_{i},{r}_{j})+\stackrel{N}{\sum_{i<j<k}}{U}_{2}({r}_{i},{r}_{j},{r}_{k})+\stackrel{N}{\sum_{i<j<k<l}}{U}_{3}({r}_{i},{r}_{j},{r}_{k},{r}_{l})+\stackrel{N}{\sum_{i<m}}{U}_{4}({r}_{i},{r}_{m})$$

where U_1_, U_2_, and U_3_ are the potential energy developed by bond stretching of covalently bonded diatomic molecules, bond angle bending of covalently bonded triatomic molecules, and bond twisting of covalently bonded tetratomic molecules, respectively. U_4_ is the potential energy corresponding to the interaction of non-covalently bonded diatomic molecules. In general, a polyatomic interaction between three or more atoms is omitted in classical molecular dynamics because it accounts for less than 10% of the total potential energy. Therefore, we approximated the potential energy of the CG models for radiation damage analysis based only on the sum of the potentials between two particles (see Fig. S2 and Eq. (2)).

2 $${U}_{total}\approx \sum {U}_{1}(r)+\sum {U}_{4}(r)$$

3 $${U}_{1}(r)={D}_{e}\{1-{e}^{-2\alpha (r-{r}_{e})}-2{e}^{-\alpha (r-{r}_{e})}\},\alpha =\sqrt{{k}_{e}/2{D}_{e}}$$

4 $${U}_{4}(r)=4{D}_{e}\{(\frac{\sigma }{r}{)}^{12}-(\frac{\sigma }{r}{)}^{6}\},\sigma =\frac{{r}_{e}}{{2}^{1/6}}$$

U_1_ in Eq. (2) was calculated using the Morse potential in Eq. (3), which adequately represents the properties of the interaction energy between covalently bonded atoms. Here, $r$ is the distance between diatomic molecules, and ${r}_{e}$ is the equilibrium bond distance. ${D}_{e}$ is the depth of the potential well that indicates the strength of covalent bonds, i.e., the dissociation energy. $\alpha$ is a parameter related to the width of the potential and can be calculated as $\alpha =\sqrt{{k}_{e}/2{D}_{e}}$. Here, ${k}_{e}$ is the force constant of the minimum of the well. The following approximate values were used depending on the bond order of diatomic molecules: 5 $\times$ 10^5^ dyn/cm for a single bond, 10 $\times$ 10^5^ dyn/cm for a double bond, and 15 $\times$ 10^5^ dyn/cm for a triple bond^31^. U_4_ in Eq. (2) was calculated using the Leonard–Jones (LJ) potential in Eq. (4), which is typically used to simulate molecules bonded by the van der Waals force. Here, $\sigma$ indicates the distance between two atoms at the point where the potential is zero. The parameters used in Eqs. (3) and (4) to calculate the CG potential are summarized in Table 1.

**Table 1:** The Morse and the Lennard–Jones (LJ) potential parameters used in this study^42,43^.

Bond	${{\varvec{r}}}_{{\varvec{e}}}$(Å)	${{\varvec{D}}}_{{\varvec{e}}}$(eV)	$\boldsymbol{\alpha }$(Å^-1^)	${{\varvec{k}}}_{{\varvec{e}}}$(dyn/cm)
C–C	1.54	3.61	2.08	5 × 10^5^ for single bond 10 × 10^5^ for double bond 15 × 10^5^ for triple bond
C–O	1.43	3.73	2.05	
O–O	1.48	1.50	3.23	
P–O	1.64	3.47	2.12	
P=O	1.50	5.64	2.35	

### Simulation configuration to validate new damage model

Figure 1d shows the simulation geometry of Geant4-DNA (version 10.7 patch 01) that was performed to verify the new damage model. The 436 bp sized plasmid fragments were positioned as daughter volumes in a cuboid mother volume with a margin of 1 nm in each of x, y, and z directions. A total of 5400 mother volumes were placed on a sphere with a radius of 3 μm to consider the same bp density as in the experiment (ρ ≃ 22.1 × 10^–5^ bp/nm^3^). The position of each mother volume was determined by generating a uniform random number. Interference between mother volumes was checked for each uniform number generation, and if interference occurred, a new random number was repeatedly generated such that there was no common interface between all mother volumes. To reflect the morphological characteristics of the dried plasmid, 90% of the total plasmids were arranged in the supercoiled form, and the remaining 10% were arranged in the relaxed form^32^.

The surface of the spherical volume was selected as the location of the primary particle. The momentum direction was defined randomly to enable uniform transmission of radiation to the plasmid segments within the sphere phantom (refer to the solid red line in Fig. 1). A wide range of LET beams were irradiated: electrons (1.0 MeV), protons (5.0 MeV, 10 MeV, 25 MeV), and alpha particles (4.0 MeV, 10.0 MeV, 20.0 MeV). It was verified that the LET of these particles ranged from 0.2 keV/μm to 99.4 keV/μm. This range was calculated based on the unrestricted linear energy transfer (LET_∞_) proposed in ICRU90^31^. The "G4EmDNAPhysics_option2" model^33,34^ was used to simulate the direct interaction of the incident radiation and plasmids. The cumulative absorption energy for each radiation event in each scoring volume was calculated based on the result. Finally, when the cumulative absorption energy of phosphate and deoxyribose scoring volumes exceeded each CG potential, a strand break (SB_break_) was considered to occur at the corresponding nucleotide location.

Once the location of the SB_break_ for each event was determined, the frequency of the SSB or DSB yield (Gy^−1^Gbp^−1^) was calculated as a function of LET using the clustering algorithm^3,7^ in Fig. S1. When multiple incidence patterns were observed, the most complex break type was selected. The SSB yield discussed in this study includes SSBs and SSB clusters (SSB^+^ and 2SSB), and the DSB yield includes DSBs and DSB clusters (DSB^+^ and DSB^++^).

### Experimental configuration to validate simulation results

The new damage model estimates the SSB and DSB yields under the direct interaction of radiation. To exclude the possibility of the effects of indirect radiation damage and *in vivo* repair factors, the dried pBR322 plasmids were irradiated with a certain dose of gamma and electron. To obtain pBR322 plasmids, *E. coli* containing the subject plasmids was cultured overnight at 37 °C and the plasmids were recovered using the “QIAprep Spin miniprep kit” provided by Qiagen. The extracted plasmids were quantified using the NanoDrop equipment. To examine the direct DNA damage owing to the radiation LET, 30 μl of plasmids at a concentration of 50 ng/μl (ρ ≃ 2.1 × 10^–5^ bp/nm^3^) were dispensed into 96 wells, freeze-dried to remove moisture, and irradiated using high-level ^60^Co (1,173 keV and 1,332 keV) and a linear electron accelerator (1 MeV) at the Korea Atomic Energy Research Institute (KAERI). To observe the morphological transformations of the plasmids depending on radiation dose, electrophoresis was performed on 10 μl (500 ng) of the irritated samples for 120 min at a voltage of 90 V in a 1% agarose gel (agarose gel electrophoresis (AGE))^35^. After electrophoresis, the agarose gel images were obtained using Gel-document system (Bio-Rad, Hercules, CA, USA). The relative intensity of DNA plasmids in each lane that manifested on the agarose gel was measured using the ImageLab program (Bio-Rad).

## Results

### Dry pBR322 plasmid irradiation

**Figure 2:**
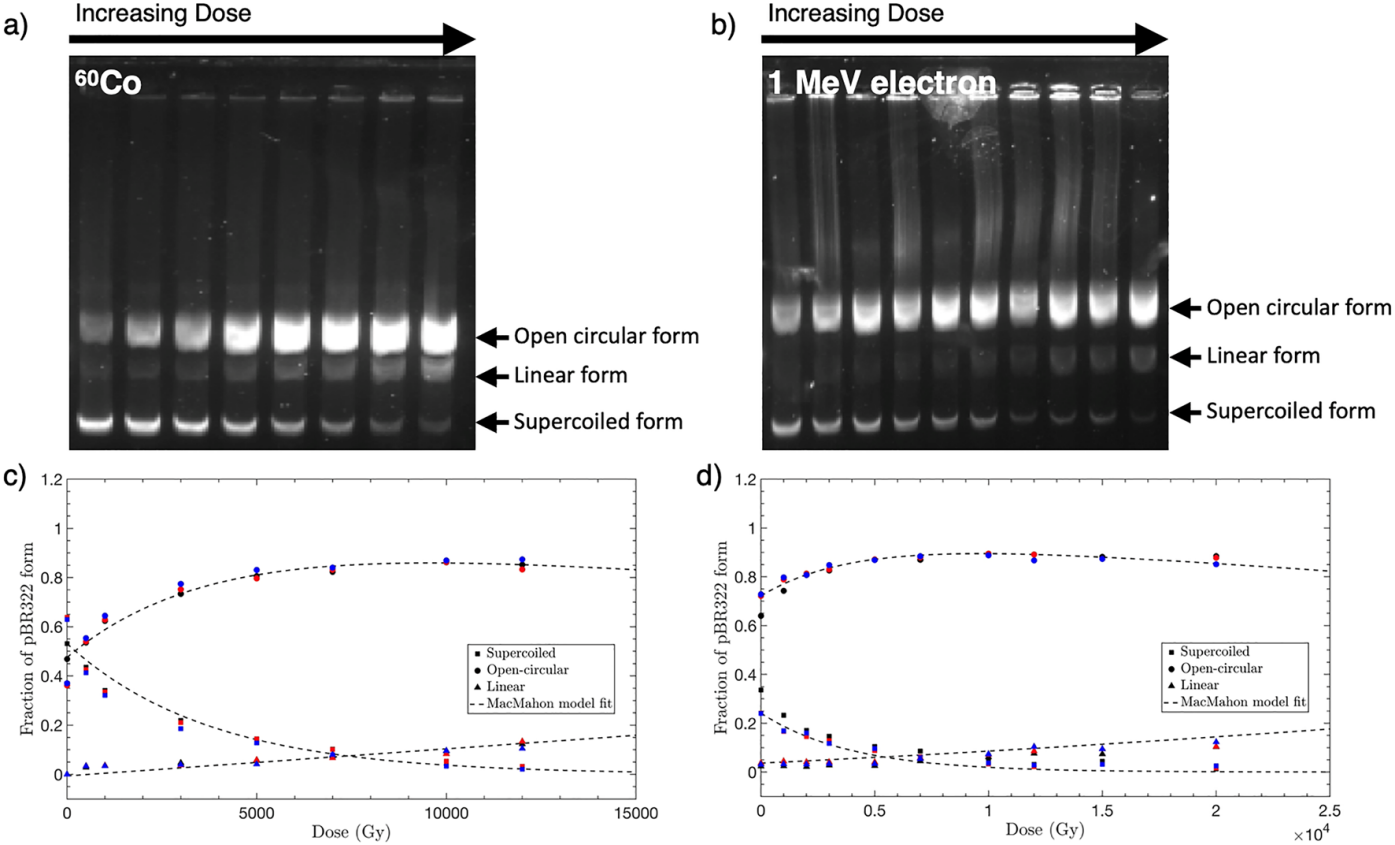
Morphological change of pBR322 plasmid and relative amounts of SC (supercoiled), OC (open-circular), and L (linear) forms for radiation dose (scatter plot) with McMahon`s model (dash-dot plot). The image of agarose gel following electrophoresis of dry pBR322 plasmid irradiated by (**a**) ^60^Co (**a**) or (**b**) 1 MeV electron irradiation (**b**). (**c**,**d**) The proportion of SC, OC, and L forms of dry pBR322 plasmid irradiated by (**c**) ^60^Co and (**d**) 1 MeV electron irradiation.

Figure 2 shows the morphological transformations of dried plasmids irradiated with ^60^Co and 1 MeV electron. It is evident that the relative intensity of the open-circular and linear bands increased with the occurrence of nicks caused by radiation exposure. In particular, as the dose was increased, the probability of nicks occurring for opposite strands increased. Therefore, the intensity of the linear form increased.

The SSB and DSB yields depending on the morphological transformations of plasmids were quantified through the McMahon fitting^36^ in Eqs. (5), (6), and (7).

5 $$SC(D)={S}_{0}{e}^{-(\mu D+\phi D)}$$

6 $$OC(D)={e}^{-\phi D}\left[{e}^{-0.5{\mu }^{2}\rho {D}^{2}}({S}_{0}+{C}_{0})-{S}_{0}{e}^{-\mu D}\right]$$

7 $$L(D)=1-({S}_{0}+{C}_{0}){e}^{-(\phi D+0.5{\mu }^{2}\rho {D}^{2})}$$

Here, SC(D), OC(D), and L(D) refer to the relative ratios of band intensities corresponding to the supercoiled, open-circular, and linear forms, respectively, after irradiation at the D(Gy). The S_0_ and C_0_ are the proportions of the supercoiled and open-circular forms, respectively, before irradiation. ρ is the probability of DSB generations owing to two SSBs on opposite DNA strands within 10 base pairs^32^. μ and φ are the average SSB and DSB yields, respectively and the parameters to be ultimately obtained through this fitting. These parameters can be obtained from all the fitting curves of Eqs. (5), (6), and (7), but according to the experimental results, R^2^ is 0.98 and 0.96 in the ^60^Co and 1 MeV datasets, respectively, and demonstrates the highest accuracy in OC(D). Therefore, the yields of SSB and DSB were derived from the OC(D) curve. Figure 2 shows the relative proportion of plasmid forms based on the radiation dose. Each color-coded scatter plot shows the independent results for three experimental datasets. Fitting was performed for each experimental dataset, and the SSB and DSB yields indicated the mean average value of each fitting result. The values of μ for ^60^Co (LET: 0.3 keV/μm) and a 1 MeV electron beam (LET: 0.25 keV/μm) were observed to be 57.4 ± 2.25 Gy^−1^Gbp^−1^ and 53.5 ± 3.3 Gy^−1^Gbp^−1^, respectively, and φ was 3.87 ± 1.21 Gy^−1^Gbp^−1^ and 1.0 ± 0.3 Gy^−1^Gbp^−1^, respectively.

### CG potential of DNA backbone

In the case of DNA, direct damage originates from phosphodiester bonds broken by energy transferred from radiation. Therefore, the potential energy for CG models corresponding to phosphate and deoxyribose was calculated. When the accumulated absorbed energy for each radiation event exceeds the potential energy, it was assumed that the binding structure of phosphate or deoxyribose was broken. This resulted in a damaged DNA backbone. Table 2 shows the results of the CG potential calculation for phosphate composed of PO_3_. The CG potential was approximately 12.4 eV, which was calculated using from the distance between the two atoms and Eqs. (3) and (4). The CG potential of deoxyribose calculated through the same process was approximately 30.5 eV.

**Table 2:** Example of the total potential energy of the phosphate Coarse-Grained model calculated from the diatomic distance of the constituent atom group.

Type	Bond	Length (Å)	Energy (eV)
Bonded	P–OP1	1.480	− 2.9038
	P=OP2	1.482	− 5.6294
	P–O5’	1.598	− 3.4339
Non− bonded	OP1–OP2	2.520	− 0.1206
	OP1–O5’	2.506	− 0.1246
	OP2–O5’	2.463	− 0.1379
U_total_			− 12.3562

### Validation of new damage model

**Figure 3:**
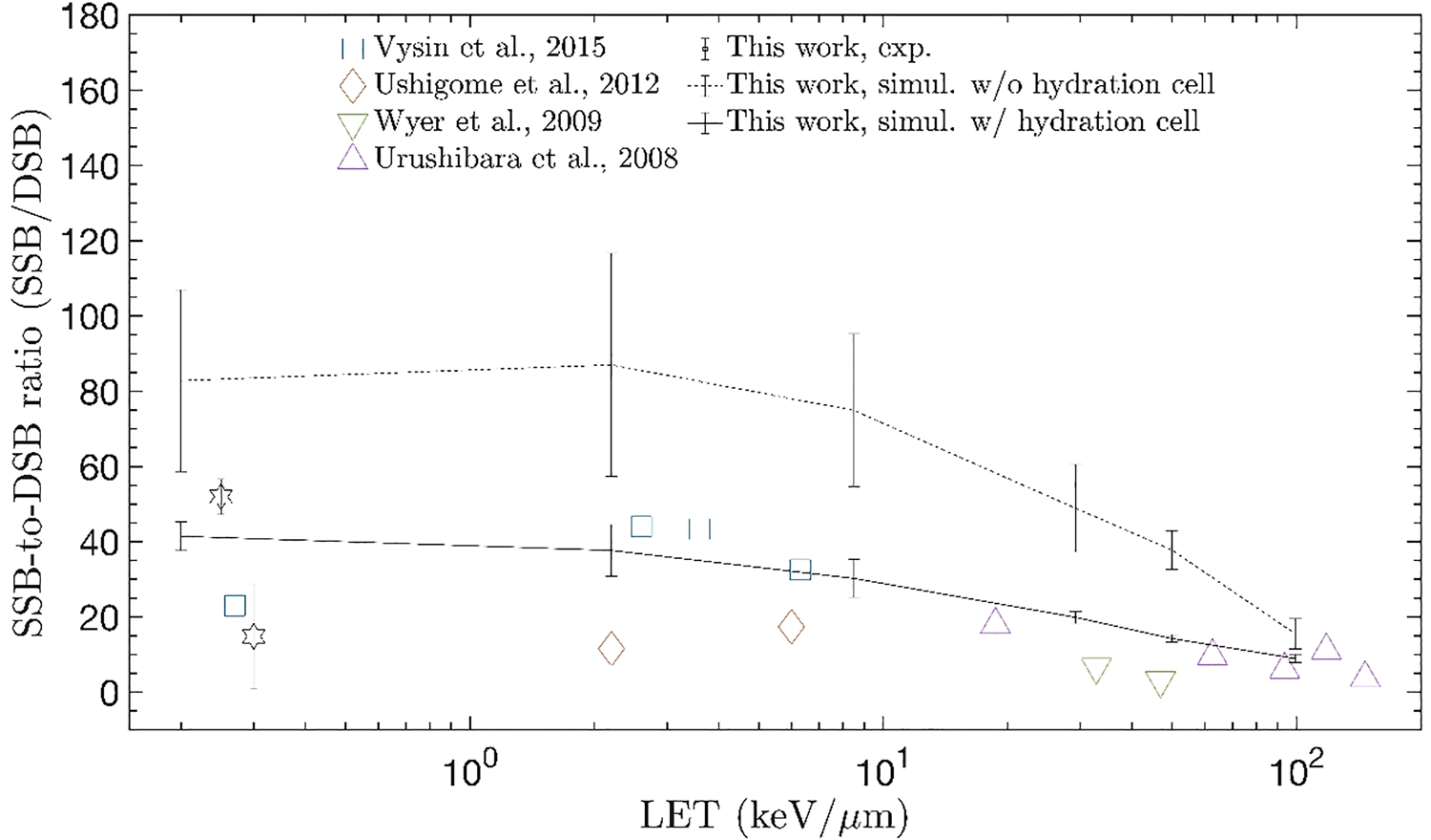
SSB/DSB ratio as a function of LET and comparison with experimental data. The scatter plot shows the experimental data, the solid-dot line shows the simulation result without considering the DNA hydration cell, and the solid line shows the simulation result considering the DNA hydration cell.

The SSB/DSB ratio in Fig. 3 is suitable for evaluating the performance of the damage model through direct comparison with measured data. This is because the effect on the uncertainty in dose calculation is eliminated. The scatter plot in Fig. 3 shows the results of the beam irradiation experiment on dried plasmids. Experimental data published by Vyšín et al.^37^, Ushigome et al.^38^, Wyer et al.^39^, and Urushibara et al.^40^ are displayed in addition to the data acquired through electron and gamma irradiation in this study. The solid-dot line indicates the simulation result when the radius of the scoring volume is defined to be VDWR as the initial condition of the damage model. The solid line represents the results after setting the radius of the scoring volume as 3.4 Å^41^ for both the phosphate and deoxyribose CG volumes. The new damage model is observed to effectively reproduce the rapidly decreasing tendency of the SSB/DSB ratio with LET of 10 keV/μm or more. However, when the radius of the scoring volume is set to VDWR, the SSB/DSB ratio is observed to be marginally higher than that of the measured data over the entire LET range. Meanwhile, the result is in better agreement with the measured data when the scoring volume is increased to 3.4 Å to consider the hydration cell of DNA. Therefore, the radius of the scoring volume was set as 3.4 Å in all the subsequent simulations.

**Figure 4:**
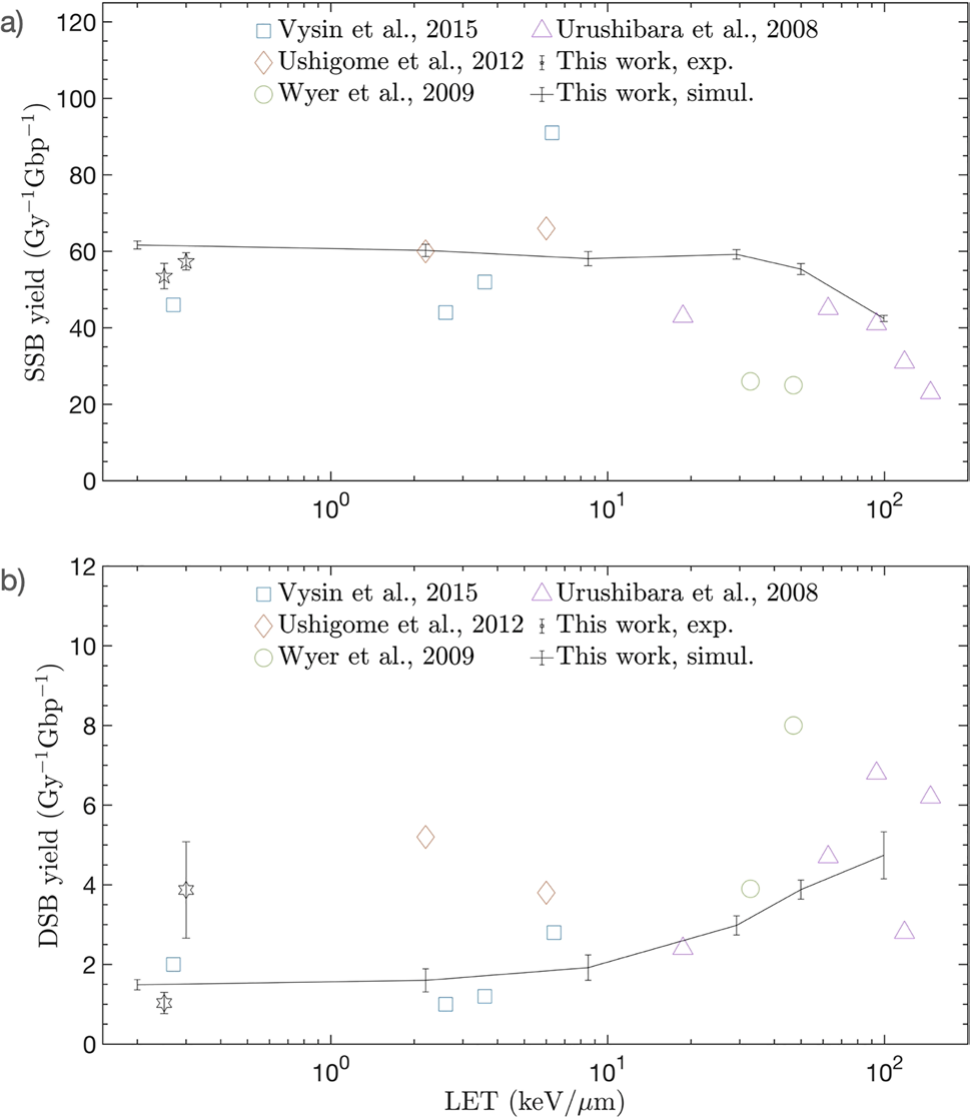
Comparison of simulation results with data from irradiation experiments for dry pBR322 plasmid. (**a**) SSB yield per Gy and per Gbp as a function of unrestricted LET. (**b**) DSB yield per Gy and per Gbp as a function of unrestricted LET.

Figure 4 shows the SSB and DSB yields as functions of LET. The new damage model effectively reproduces the SSB yield, which is weakly dependent for LET below 50 keV/μm. At a higher LET, the result is consistent with the experimental data in which SSB decreases when DSB increases. This is because high LET particles are more likely to generate SB_break_ that is close enough (≤ 10 bp) to induce DSB owing to increased clustering. For a quantitative evaluation of the new damage model, we calculated the mean percentage error from the experimental data using the following Eq. ^9^:

8 $$\stackrel{n}{\sum_{i=1}}\mid ({V}_{i}^{sim}-{V}_{i}^{exp})/{V}_{i}^{exp}\mid /{n}_{data}\times 100$$

Here, ${V}_{i}^{sim}$ is the i-th simulation value, and ${V}_{i}^{exp}$ is the i-th experiential value. ${n}_{data}$ refers to the number of experimental data. The new damage model was observed to replicate the measured data within an error range of approximately 14.2% over the entire LET range.

**Figure 5:**
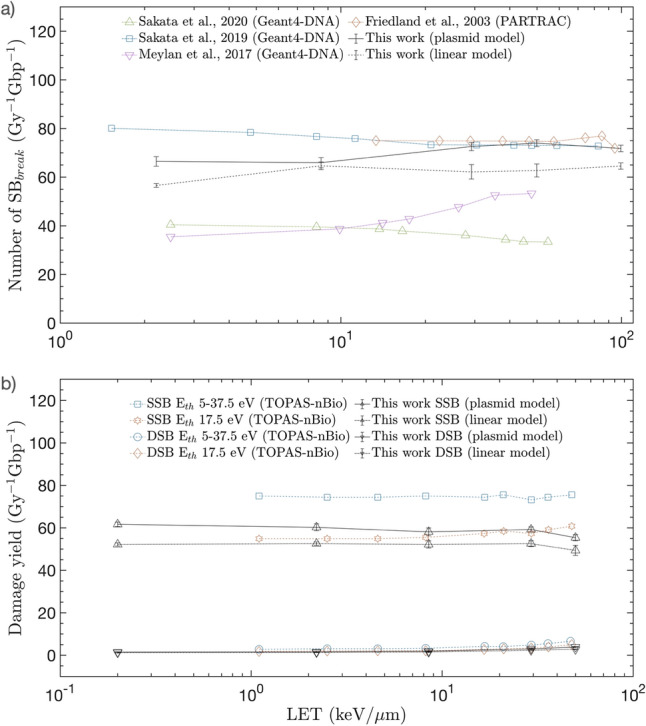
Radiation direct damage results for plasmid and linear DNA models and comparison with the previous simulations. (**a**) Number of direct strand breaks (SB_break_) per Gy and Gbp as functions of unrestricted LET. (**b**) Damage yield per Gy and Gbp as function of unrestricted LET and comparison with TOPAS-nBio results in accordance with the threshold energy.

Figure 5 shows the simulation results performed to confirm that the new damage model is also applicable to linear type DNA and comparison with previous simulation results^6,9,14,15,23^ calculated with the parameters in Table 3. Linear DNA simulations were performed using the same procedure as for plasmid simulations. However, the size of the linear DNA was 146, and a total of 2,362,500 DNA segments were placed on a sphere with a radius of 3 μm to maintain the bp density. SB_break_ of Fig. 5a is observed to increase slightly as LET increased in both linear and plasmid model. This can be explained as the cumulative energy in a given target per unit of absorbed dose that increases with LET. However, while the LET increases by approximately 500 times from 0.2 to 99.4 keV/ μm, the change in SB_break_ is only approximately less than 15%, thereby it was reconfirmed the results of previous simulations that the SB_break_ caused by direct radiation damage is not significantly dependent on LET. Figure 5b shows the comparison of the results of the TOPAS-nBio^14^, which calculates the difference in damage to the threshold energy, with the results of the new damage model. It can be conformed that the new damage model is comparable to the TOPAS-nBio results calculated using a threshold energy of 17.5 eV. In the LET region of less than 100 keV/ μm, both plasmid and linear models showed comparable results, demonstrating that damage model for estimating radiation damage to DNA without empirical data was possible.

**Table 3:** Direct-damage parameters of this work and previous Monte Carlo simulations.

Parameter	Friedland (2003)	Meylan (2017)	Sakata (2019)	Sakata (2020)	This work
*R*_dir_ (Å)	2VDWR	VDWR	4.5	3.5	3.4
${{E}_{min}^{break}}(eV)$	5	17.5	5	5	12.4@phosphate 30.5@deoxyribose
${{E}_{max}^{break}}(eV)$	37.5	17.5	37.5	37.5	12.4@phosphate 30.5@deoxyribose

${R}_{dir}$ Accumulation radius of energy deposition from nucleotide center, ${E}_{min}^{break}$ Minimum energy of direct strand break probability, ${E}_{max}^{break}$ Maximum energy of direct strand break probability model, *VDWR* Summation of atomic volume with each atomic van der Waals radius.

## Discussion

In this work, we aimed to construct a model for radiation damage analysis based on the "first principle method" ultimately pursued in simulation. First principle implies that the performance of calculations are completely independent of empirical quantities. To achieve this, we presented a new damage model that could approximate simulation parameters without experimental data. In this study, data were acquired through irradiation experiments on dried pBR322 plasmids to suppress the effects of indirect damage caused by radicals and *in vivo* repair factors. Furthermore, the new damage model for estimating direct radiation damage was validated by comparing the experimental data with simulation data under identical conditions.

In the new damage model, the minimum repeating units of biomolecules were grouped in a bead to implement CG models and were used as a unit volume for calculating the cumulative absorption energy of radiation. In addition, for the first time, we proposed a method of calculating potential energy based on the bonding structure of the atomic clusters constituting the CG models, and using it as the threshold energy. When the new damage model was applied to DNA (a typical biomolecular system), and CG models could be constructed with phosphate, deoxyribose, and base as the minimum repeating units. The CG potentials of phosphate and deoxyribose were calculated considering that the direct radiation damage to DNA was caused by the breaking of phosphodiester bonds.

Phosphate groups usually consist of PO_4_ (P, OP1, OP2, O3’, O5’). Of these, O3’ and O5’ are covalently bonded to C3’ and C5’ of the deoxyribose group, respectively. If the boundary of the CG volume is defined, O3' and O5' can belong to either the phosphate or the deoxyribose groups. Therefore, considering the fairness of the binding energy calculation, O5' and O3' were subordinated to the phosphate and deoxyribose groups, respectively. Because the phosphate group was composed of PO_3_ (P, OP1, OP2, O5’), the calculated binding energy was approximately 3 eV lower than in the general case. Moreover, the reduced binding energy was added to the potential energy of the deoxyribose group by the O3’-C3’ bond. This damage model assumed that the nucleotide is damaged when the cumulative radiation energy exceeded the potential in one or both volumes of the phosphate and deoxyribose. Therefore, the potential energy of 3 eV, which can be decreased or increased by the CG volume boundary, can cancel each other from the viewpoint of one nucleotide, and do not significantly affect the overall simulation results. The CG potentials of phosphate composed of PO_3_ and deoxyribose composed of C_5_O_2_ were calculated to be 12.4 eV and 30.5 eV, respectively. These values fall in the range of the experimental data-based threshold energy (5–37.5 eV) used in existing simulations. This verifies that the threshold energy for radiation damage could be approximated without empirical quantities.

The SSB/DSB ratio results in Fig. 3 verify that the new damage model effectively replicated the decreasing tendency of SSB/DSB when an increase in the LET is observed in the experimental data. However, the simulation result was observed to be marginally higher than the measured data when the radius of the CG model was set to VDWR. Schneider et al.^41^ have reported that in the double helix DNA structure, four or five water molecules per nucleotide constitute the first hydration cell within 3.40 Å from a phosphate or base atom. We considered this to supplement the results of the new damage model. By adjusting the CG radius for phosphate and deoxyribose from VDWR to 3.40 Å, we could obtain results that were closer to the measured data. The results from the study verified that the charge transfer effect caused by the hydration cell should be reflected in the radiation damage simulation of DNA. The results also verified that a realistic geometry is necessary to increase the reliability of the radiation damage simulation results of biomolecular systems.

Figure 4 illustrates a comparison of the SSB and DSB yields determined as functions of LET, with the experimental data. For LET particles in the range of 0.2–99.4 keV/µm, the damage model reproduced the measured data with an error of approximately 14.2%. In a previous simulation study^7^, the systematic uncertainty from a physical particle transport model was estimated to be approximately 20% for the electron-induced DSB yield of simple DNA fibers. Similarly, according to Zhu et al.^14^, the physical model could result in uncertainty of up to 34% in a DSB yield simulation of a cell nucleus model. It was also reported that the direct damage model resulted in a divergence of at most 28% from the measured data^14^. Considering the results of these prior studies, the new damage model appeared to reproduce experimental data adequately and demonstrated its effectiveness in predicting direct radiation damage to DNA.

The study of radiation damage simulation for biomolecular systems is a process of identifying the best approximation method while maximizing accuracy, based on valid physical evidence. In this study, we proposed a new method to approximate simulation parameters without using empirical data by applying the concept of CG potentials commonly used in molecular dynamics, to existing Monte Carlo radiation simulation. Furthermore, we demonstrated its effectiveness through experimental validation. The new damage model would be highly applicable for predicting damage to living organisms caused by radiation exposure, which is required for radiation protection in fields such as medicine, nuclear energy, and space exploration.

## Conclusion

In this study, we proposed a new damage model that could be applied in radiation damage models for biomolecules and investigated its application in the Geant4-DNA simulation. The key aspect differentiating the proposed model from existing models is that the potential energy of CG models is replaced with the threshold energy for radiation damage. The potential energy of CG models reflects the bonding structure of the constituent clusters. Therefore, we assumed that the transfer of energy exceeding the potential energy would affect some of the bonding structures and result in radiation damage such as cleavage. It is moderately different from the typical method of using the ionization energy of atoms as threshold energy. To examine the effectiveness of the method, the Geant4-DNA simulation was performed on pBR322 plasmids and was compared to measured data from the perspective of direct radiation damage. The method proposed in this study was capable of replicating the experimental measurement with an error of 14.2% with LET ranging from 0.2–99.4 keV/µm. This verified its validity as a new radiation damage model.

## Supplementary Information

Supplementary Information.

## Data Availability

All data generated or analyzed during this study are included in the article and supplementary information. The source code used in this study are available from the corresponding author through a reasonable written request.
