# Supplementary material for: New damage model for simulating radiation-induced direct damage to biomolecular systems and experimental validation using pBR322 plasmid
Source: Sci Rep. 2022 Jul 5;12:11345. doi: 10.1038/s41598-022-15521-y (PMC9256689; doi:10.1038/s41598-022-15521-y)
Supplement: Supplementary file 1 — Supplementary Information. [file 41598_2022_15521_MOESM1_ESM.docx]

**New damage model for estimating radiation-induced direct damage to biomolecular systems: Geant4-DNA application and experimental validation using pBR322 plasmid**

Jinhyung Park^1^, Kwang Woo Jung^1^, Min Kyu Kim^1^, Hui-Jeong Gwon^1^ and Jong Hyun Jung^1*^

^1^Advanced Radiation Technology Institute, Korea Atomic Energy Research Institute, Jeongeup 56212, Republic of Korea.

^*^Corresponding: jungjh83@kaeri.re.kr


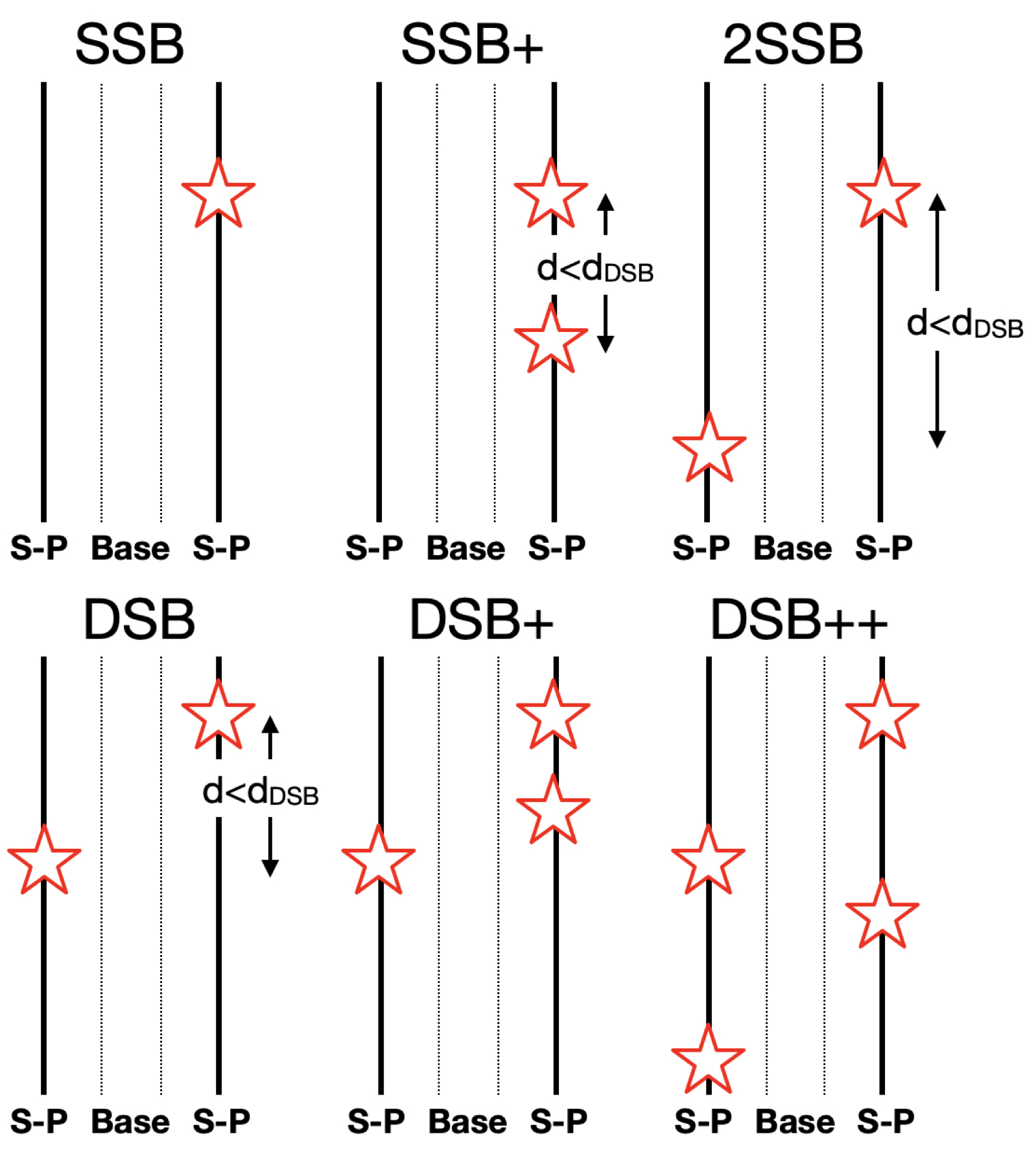


**Supple Figure S1**. The scheme of classification for cluster damage of SSB and DSB


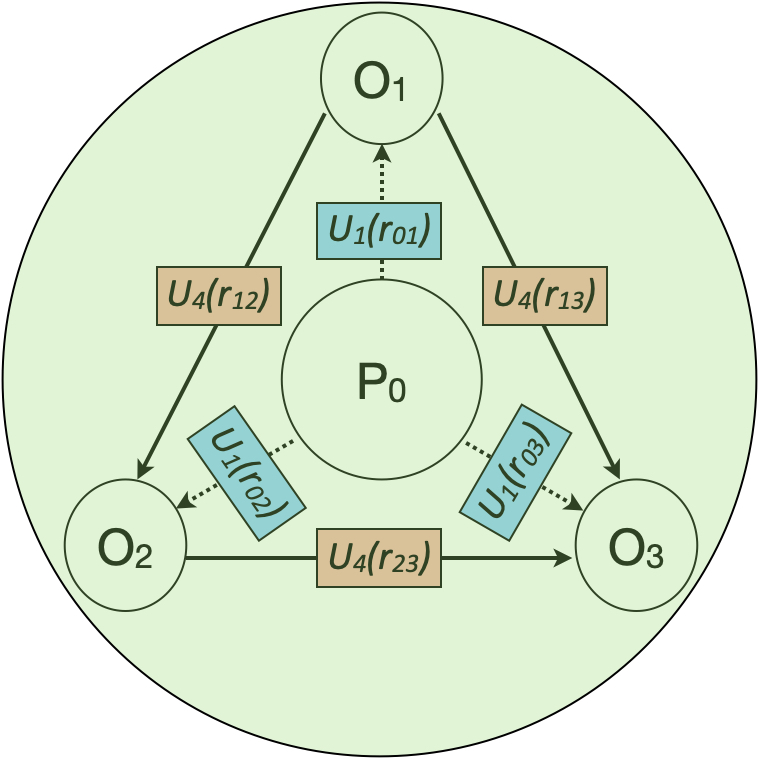


**Supple Figure S2**. Example of pairwise interactions between phosphate (PO_3_) constituent atoms.
